# Perceptions and Attitudes of Patients and Health Care Stakeholders on Implementing a Telehealth Service for Preoperative Evaluation: A Qualitative Analysis

**DOI:** 10.1089/tmr.2023.0023

**Published:** 2023-06-26

**Authors:** Eileen Lew, Sean F.J. Tan, Agnes Teo, Ban L. Sng, Elaine P.M. Lum

**Affiliations:** ^1^ Women's Anaesthesia, KK Women's and Children's Hospital, Singapore, Singapore.; ^2^ Anaesthesiology and Perioperative Sciences Academic Clinical Program, SingHealth Duke-NUS Academic Medical Centre, Singapore, Singapore.; ^3^ Lee Kong Chian School of Medicine, Nanyang Technological University, Singapore, Singapore.; ^4^ Health Services and Systems Research, Duke-NUS Medical School, National University of Singapore, Singapore, Singapore.

**Keywords:** telemedicine, telehealth, preoperative evaluation, normalization process theory, implementation science

## Abstract

**Background:**

Studies suggest that preoperative evaluation can be effectively conducted through telehealth. As the COVID-19 pandemic has accelerated digital transformation, we hypothesize that a new telehealth model of care may be feasibly implemented for preoperative evaluation at our institution. This qualitative study seeks to evaluate the attitudes and perception of elective surgery patients and health care providers toward telehealth conducted for preanesthesia evaluation.

**Methods:**

At a tertiary women's hospital in Asia, health care providers and elective surgery patients were recruited by convenience and snowball sampling to undergo one-on-one semistructured interviews regarding a new telehealth model of care for preanesthesia evaluation, under-pinned by the Normalization Process Theory. Data were analyzed, coded, and consolidated into themes using the framework analysis method by a team of four researchers from diverse backgrounds.

**Results:**

Twenty-five interviews were conducted among 10 patients and 15 health care participants. Ninety-five codes were identified, consolidated into four themes that connect to guide the implementation of a new telehealth pathway for preoperative care, mapped to the Normalization Process Theory. The themes pertain to advantages of telehealth workflow (coherence), requisites for new telehealth workflow (coherence, collective action), barriers to implementation (cognitive participation, collective action), and enablers of implementation (cognitive participation, collective action). All participants were receptive to telehealth, but health care participants expressed concern about the impact of additional tasks on current clinical workload. Training in videoconferencing was deemed essential by both patients and health care providers.

**Conclusions:**

The study has provided insights into levels of coherence and cognitive participation among patients and health care providers. The telehealth workflow should be redesigned, considering systems' constraints and stakeholders' needs. Greater buy-in is needed to gain health care providers' commitment for collective action. Clinicaltrials.gov identifier: NCT05781789

## Introduction

Preoperative evaluation is an integral part of providing anesthesia care. Failure to conduct timely preoperative evaluation contributes to increased perioperative complications,^1,2^ preoperative delays, and last-minute case cancellations.^3–5^ Preoperative evaluation is usually conducted by an anesthetist or nurse practitioner at an outpatient facility, days to weeks before the scheduled surgery. The 2019 coronavirus pandemic (COVID-19) caused an unprecedented disruption to health care services worldwide and accelerated the adoption of technology,^6,7^ with telehealth being hailed a viable alternative to in-person consultations.^8^ Consequently, there has been a surge in telehealth initiatives as a means to ensure continuity of care amidst a pandemic.

Telehealth or telemedicine has been defined as the provision of health care services using information and communication technologies, where distance is a barrier.^9^ A growing body of evidence suggests that telehealth could facilitate remote preoperative evaluation. Since the first reported case of preoperative evaluation conducted through telehealth in 2004,^10^ studies have shown high levels of satisfaction experienced by both patients and health care providers.^10–13^ For patients, telehealth eliminates unnecessary travel, and saves time and costs.^14,15^ Importantly, preoperative evaluation through telehealth is as effective as in-person assessment, with comparable surgical cancellation rates.^12,16,17^ However, adoption of telehealth has remained slow and variable across all domains of medicine until recently.

In line with global trends, we hypothesize that a new telehealth model of care for preoperative evaluation could be feasibly implemented and accepted by stakeholders at our institution. If successfully implemented, we anticipate that >90% of our patients scheduled for elective surgery could safely undergo preoperative evaluation through telehealth. As telehealth is not a mainstream model of care locally, we anticipate that willingness to participate in virtual consultations may not be universal for both patients and health care providers.

The aim of our study is to explore the perceptions and attitudes of patients and health care providers toward a new telehealth model of care using semistructured interviews, underpinned by the Normalization Process Theory (NPT), which proposes that the work of implementation is done through four generative domains (coherence, cognitive participation, collective action, and reflexive monitoring) that are, in turn, influenced by factors that promote or inhibit the integration and embedding of complex interventions into routine practice.^18,19^

## Methods

### Study setting

The study was conducted at the KK Women's and Children's Hospital (KKH)—the largest hospital within Singapore that specializes in tertiary care for women and children. The Preadmission Service (PAS) at KKH attends to about 250 same-day-admission (SDA) surgical patients every month. Aided by a preanesthesia health screening questionnaire developed and validated internally,^20,21^ trained nurses perform the first triage to identify patients who require an anesthetist's assessment 2–4 weeks before surgery. Patients not requiring referral to the anesthetist undergo preoperative evaluation on the day of surgery, whereas patients with medical issues are flagged up for in-person assessment at the PAS. This process has kept the institution's surgical case cancellation rates for elective surgery at about 5% (unpublished data).

### Study design

We used a sequential exploratory mixed methods study design. An implementation science framework—the NPT- underpins our 3-phase study where Phase 1 (service redesign) explores the feasibility of presurgery telehealth through semistructured interviews of patients and health care providers; Phase 2 (preimplementation) investigates efficacy of the telehealth model of care by addressing service fidelity, and patient and health care provider experience using administrative data; Phase 3 is a pragmatic trial to ascertain uptake and cost-savings of the telehealth model of care. This article focuses on Phase 1—service redesign, in which the following research questions (RQ) were posed:

RQ1. How feasible is telehealth preoperative evaluation for patients and health care providers in our health care setting?

RQ2. How acceptable is the concept of telehealth preoperative evaluation to patients and health care providers?

To address these RQ, we conducted one-on-one semistructured interviews with patients and health care providers at the surgeons' clinics and the PAS. Interview guides were formulated for health care providers and for patients, underpinned by the NPT (Supplementary File SX). Four NPT domains characterize the work needed to be done in implementing and embedding a change into routine practice—in this case, telehealth for preanesthesia evaluation. The four domains are *sense-making* or *coherence*, *cognitive participation*, *collective action*, and *reflexive monitoring.*^18,19^

This study teases out the domains of *coherence, cognitive participation*, and *collective action*, where *coherence* is the understanding of how telehealth differs from conventional practice of in-person evaluation, and the value of telehealth. *Cognitive participation* encompasses the roles of health care providers and patients in the new telehealth workflow, including factors that promote or inhibit participation. *Collective action* is the work of health care providers in operationalizing the telehealth workflow and is mediated by factors such as skill sets, contextual integration, and the relationships needed to drive the change forward. *Reflexive monitoring* is the formal and informal appraisal work that people do to judge the effects of an implementation effort.^19^

### Recruitment of participants

Hardcopy and e-brochures were disseminated 2 weeks before study initiation to create awareness at the surgery clinics and PAS. We aimed to recruit 15 health care providers and 10 patient participants through convenience and snowball sampling, with the intent to continue sampling until theoretical saturation.^22,23^ All patients scheduled for SDA surgery and conversant in English were invited to participate at the PAS. Health care participants were recruited from their respective clinics, targeting surgeons, anesthetists, nursing staff, and clinic managers, whose perceptions and concerns would shape the new telehealth workflow. Written informed consent was obtained before the interview. All patient and health care participants who were approached consented to the participation and all participants completed the interviews.

### Interview process

**FIG. 1.**
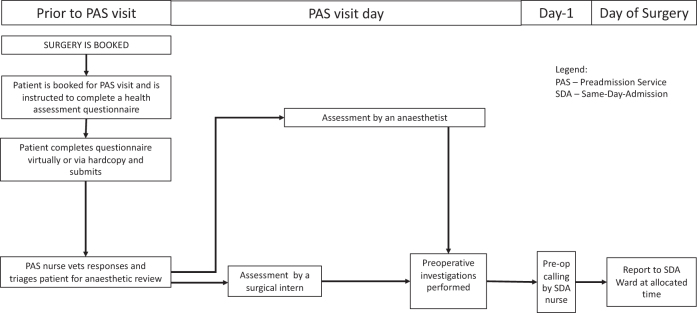
Current workflow for elective SDA surgery patients.

**FIG. 2.**
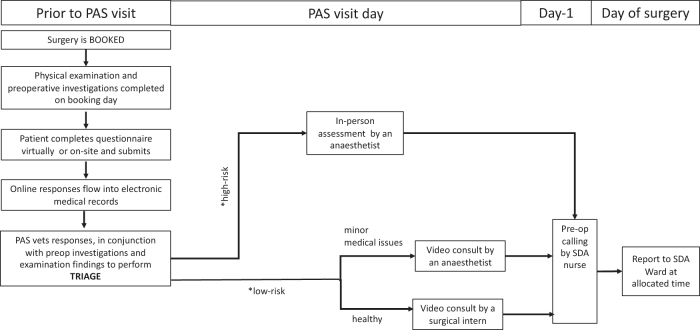
New telehealth workflow for elective SDA surgery patients.

All interviews were conducted face-to-face between July and November 2021 and audio-recorded. The researcher explained the purpose of the study and showed pictorial representations of the current and proposed new telehealth workflows (Figs. 1 and 2) for clarity. Any questions about the study were addressed before starting the interview.

Patients were interviewed in a private consultation room at the PAS. Health care participants were interviewed in a quiet meeting room in the hospital. Patient participants who completed the one-on-one interviews were provided with reimbursement for transport. Health care participants were not reimbursed.

Interviews were conducted by two authors (E.L. and A.T.). Health care participant interviews were conducted by a consultant anesthetist (E.L.), whereas patient interviews were conducted by a trained clinical research coordinator (A.T.). Both researchers have deep experience and knowledge in preoperative clinical workflows, allowing them to understand participant viewpoints and to engage in fruitful in-depth conversations. The recorded interviews were conducted in a conversational informal manner with paraphrasing and clarifications to increase the accuracy of understanding. Interview guides were used (Supplementary File SX), but interviewers stayed attentive to nuanced responses to pursue fruitful lines of questioning.

### Data analysis

Audio recordings of the interviews were transcribed verbatim by a third-party vendor and checked for accuracy (A.T.). We used the framework analysis method^24^ to analyze the data. Before the inductive coding of each transcript, researchers first familiarized themselves with the transcripts.

To ensure trustworthiness, three researchers (E.L., A.T., and S.F.J.T.) independently and inductively coded three transcripts randomly selected from 15 interviews of health care participants. We critically compared our codes, and discussed and refined these codes after each transcript was coded. We reflexively made coding decisions contextualized by our prior experience and questioned our biases and assumptions. We sought alternative perspectives made possible by the diversity of our research team, which comprised senior doctors (B.L.S. and E.L.), a health services researcher and pharmacist (E.P.M.L.), a senior health care research associate (A.T.), and a medical student (S.F.J.T.). After consensus discussions (E.L., A.T., S.F.J.T., and E.P.M.L.), we created our initial coding framework.

The remaining transcripts were then randomly distributed and independently coded (E.L., A.T., and S.F.J.T.) using the initial coding framework. We met for discussions after each had coded a further two transcripts: to ascertain the applicability of the framework, to ensure continued consistency in coding, to surface any new codes or need for further refinements to the coding framework. Revisions to codes were shared with the research team and were accepted after discussion. The revised coding framework was then used to code the remaining transcripts; refinements were made iteratively. The same coding framework was then used to deductively code transcripts of patient interviews. Inductive codes from patient interviews were also added to the coding framework. The final coding framework is provided as Supplementary File SY. Codes were then iteratively grouped into themes. We maintained an audit trail consisting of research decisions and meeting minutes.

### Research ethics

Ethics approval for this study (CIRB 2021/2212) was provided by the SingHealth Centralised Institutional Review Board D on May 27, 2021.

## Results

During the study period, 25 interviews were conducted among 10 patients and 15 health care providers. Data saturation was attained after the 12th participant for health care provider interviews and after the 7th participant for patient interviews. To confirm data saturation, we conducted six additional interviews, three each for health care and patient participants.

Participant characteristics are shown in Tables 1 and 2. Health care participants comprise patient service associates, clinic nurses, nurse managers, and an administrator. Key stakeholders of the proposed new workflow were included in the study. Duration of interviews with health care participants ranged from 11 min to 25 min 42 sec (median 17 min 26 sec).

**Table 1.** Characteristics of Health Care Participants

*Health care participants*	*^a^Specialty or service*	*Years in practice*
Staff 1	PAS	10
Staff 2	Administrator	19
Staff 3	SAS	1.5
Staff 4	PAS	7
Staff 5	SAS	12
Staff 6	Anesthesiology	6
Staff 7	OBGYN	20
Staff 8	OBGYN	11
Staff 9	Anesthesiology	9
Staff 10	SAS	31
Staff 11	Nursing	27
Staff 12	Nursing	24
Staff 13	OBGYN	7
Staff 14	OBGYN	10
Staff 15	PAS	20

^a^ Surgery clinics at our institution are under the charge of SAS.

OBGYN, Obstetric and Gynecological Service; PAS, Preadmission Service; SAS, Specialty and Ambulatory Service.

**Table 2.** Characteristics of Patient Participants

*Patient participants^a^*	*Age (years)*	*Type of surgery*
Patient 1	45	Hysteroscopy, dilatation and curettage, polypectomy
Patient 2	49	Hysteroscopy, dilatation and curettage, polypectomy
Patient 3	39	Open myomectomy, hysteroscopy
Patient 4	35	Laser cone biopsy, IMPLANON™ insertion
Patient 5	44	Hysteroscopy, dilatation and curettage, polypectomy
Patient 6	33	Hysteroscopy, dilatation, and curettage
Patient 7	48	Total abdominal hysterectomy, right salpingo-oophorectomy, left salpingectomy
Patient 8	37	Open myomectomy
Patient 9	30	Open myomectomy
Patient 10	46	Total laparoscopic hysterectomy, bilateral salpingectomy

^a^ All female as our institution is a women's hospital.

Patient participants were all female, aged between 30 and 49 years, and scheduled for gynecological surgeries (Table 2). As our institution is a women's hospital, this sample is representative of the profile of SDA patients seen at the PAS. Patient interviews ranged from 8 min 6 sec to 13 min 48 sec (median 9 min 45 sec).

**FIG. 3.**
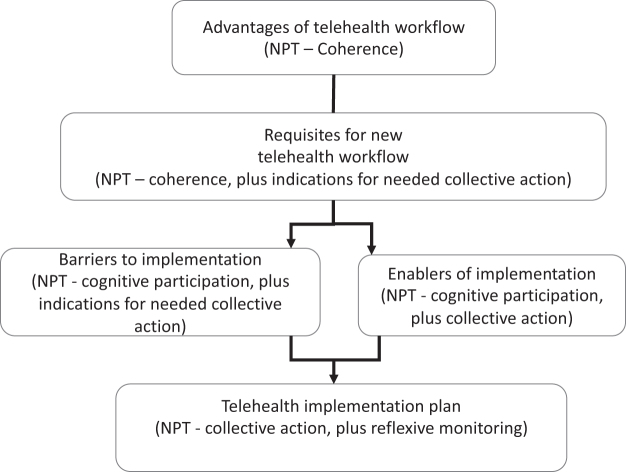
How themes potentially connect together to guide implementation.

Ninety-five codes were identified, consolidated into four themes that connect together to guide the implementation of a new telehealth pathway for presurgery care, mapped to relevant domains of the NPT (Fig. 3). Table 3 shows the four themes and accompanying subthemes identified from the data: (a) advantages of telehealth workflow, (b) requisites for new telehealth workflow, (c) barriers to implementation, and (d) enablers of implementation.

**Table 3.** Themes and Subthemes Obtained from Qualitative Analysis

*Themes*	*Subthemes*	*Indicative quotes*
Advantages of telehealth workflow	• Patient convenience • Reduced risk of exposure to COVID-19 virus • Optimizing resources for patient care	“I have to bring my children… so it helps if you can do [consults] from home” (Patient 8, age 37 years) “We don't need to [be in] so-called close contact with the patient” (Staff 4, PAS). “Doctors will probably have more time to … focus on the high-risk patients… I think it's a very good triaging system” (Staff 8, OBGYN).
Requisite for new telehealth workflow	• Physical examination must be performed on day of booking for surgery. • Preoperative investigations must be completed on day of booking for surgery. • Robust triaging criteria	“I do not listen to the lungs… [or] auscultate the heart …[if the patient] seems well” (Staff 7, OBGYN). “I foresee an issue because … there is a time lapse between the time of listing of the patient to surgery … [results may] not …be valid” (Staff 7, OBGYN). “If we put in place very clear … robust guidelines … we will be able to cater to majority of the patients' needs” (Staff 6, Anesthesiology).
Barriers to implementation	• Challenges in the use of telehealth platforms • Limitations of telehealth • Low uptake of online preanesthesia health assessment	“[Some patients] may not be able to handle the computer…especially the old folks … some old folks may not be able to read English” (Staff 10, SAS). “[If doctors] need to physically examine the patients or … require any physical contact, then telehealth would not be suitable” (Staff 15, PAS). “Talking to patients, sensing their needs online could …actually be very different from … face-to-face” (Staff 6, Anesthesiology). “… how much are [patients] able to give a comprehensive answer? Whether or not it will corroborate [with the] medical history” (Staff 13, OBGYN).
Enablers for implementation	• Skills and resources • Receptiveness toward the new telehealth workflow	“Talking to patients, sensing their needs online could actually be very different from a face to face or through a telephone. So, this could be some special skillsets that we actually may need to train our staff [in]” (Staff 6, Anesthesiology). “if [staff are] being trained, they can…. they like to teleconference with [patients] and family members” (Staff 1, PAS)

**a. Advantages of telehealth workflow**

Participants described key advantages anticipated in the proposed telehealth workflow.

### Patient convenience

All patient participants were in favor of the new telehealth workflow due to the perceived convenience. One patient declares that it “saves us time from coming down” (Patient 3, age 39 years), whereas another feels it helps to “save my leave “from work (Patient 4, age 35 years). Telehealth also allows patients to manage their childcare responsibilities while receiving consults (Table 3). Healthcare participants shared similar beliefs: “Now a lot of people [are] working from home … [if] you give them a [video consult], they don't need to travel” (Staff 10, SAS).

### Reduced risk of exposure to COVID-19 virus

Patients and health care participants acknowledged that telehealth reduces the risk of exposure to the Covid-19 virus. “Nobody really wants to …. go out so often nowadays, … with COVID cases so high now. So, with this, … [it] reduces unnecessary travel …” (Patient 3, age 39 years). Health care participants also value the reduced exposure to infectious risks (Table 3).

### Optimizing resources for patient care

Health care participants also recognized benefits of the triaging system in allocating resources to high-risk patients who are evaluated in-person (Table 3).

Furthermore, as the new workflow requires preoperative investigations to be completed at the time of booking, early detection of clinical abnormalities may circumvent cancellations on the day of surgery. “This workflow actually initiates investigations to be done earlier than what we usually do now…[where] abnormal investigations are only … highlighted after [patients have] already been seen at the PAS” (Staff 9, Anesthesiology).

**b. Requisites for the new telehealth workflow**

We identified key requisites for the telehealth workflow, outlined in the following three subthemes (Table 3).

### Physical examination must be performed on the day of booking for surgery

As patients will not undergo physical examination in a video consult, health care participants were interviewed regarding the feasibility of performing heart and lung examinations on surgery booking day. Although health care participants acknowledged the importance of performing “[auscultation] of the heart and lungs… preliminary assessment of dental [hygiene]” (Staff 6, Anesthesiology), there was ambivalence in completing that at the surgeon's clinic. One health care participant admitted that physical examination was not routinely practiced at the surgeon's clinic (Table 3). Others cited concerns of time pressures and potentially missing a diagnosis if examination is performed by staff who may not do it routinely (Staff 14, OBGYN). Others thought that surgeons could be persuaded: “I feel the basic heart, lung and abdominal examination shouldn't be a problem… [and surgeons] could be convinced to do it” (Staff 13, OBGYN).

When asked to brainstorm alternative workflows, there were suggestions to upskill nurses. “Can we [*sic*] upskill ….Advanced Practice Nurses or specialty nurses?” (Staff 2, Administrator).

### Preoperative investigations must be completed on day of booking for surgery

Before telehealth consults, patients would also need to undergo preoperative testing on the day of booking for surgery. This elicited concerns of adding burden to the current clinical load in surgeons' clinics, where nurses are already multi-tasking “…taking blood, giving medications…” (Staff 4, PAS).

There were also concerns that preoperative test results would no longer be valid on the day of surgery, given that the interval between booking and surgery could be more than 8 weeks and current accepted validity of preoperative testing is 4 weeks (Table 3).

To address the capacity issue, health care participants suggested that mobile phlebotomists could be deployed between PAS and surgeon's clinics, based on clinical load. Another health care participant wondered if patients could have preoperative testing done at a primary care facility located near their homes: “Investigations can be done in the general practice setting …. But… it's still a separate visit to another institution” (Staff 13, OBGYN). There were also suggestions for PAS to accept patients as unscheduled walk-ins on the day of booking.

### Robust triaging criteria

Several health care participants emphasized that having well-crafted robust criteria are integral to the implementation of the telehealth workflow (Table 3).

**c. Barriers to implementation**

Patient and health care participants highlighted potential barriers to telehealth implementation.

### Challenges in the use of telehealth platforms

Video consultations may not be feasible for patients who lack the digital literacy and equipment (Table 3). Some health care participants felt that patients might be less receptive to video consults as they believed video consults to be less thorough than physical consults. A health care participant expressed, “I'm not quite sure if [patients] will feel that their care will be compromised [with telehealth]” (Staff 11, Nursing).

Both patient and health care participants also cited factors that could negatively impact the telehealth experience, namely poor Wi-Fi connectivity and technical issues during log-in. Training in the use of videoconferencing was deemed important for both patients and health care providers.

### Limitations of telehealth

Video consultations would immediately preclude patients who require physical examination for the diagnosis of underlying conditions (Table 3). Another health care participant lamented: “[With the patient] behind the screen… [it's hard to tell] what's wrong with the patient” (Staff 4, PAS). Others felt that this could be addressed, in part, by good lighting and the use of higher-resolution video consultation platforms, for example, in the examination of the airway. Notably, patient participants expressed the wish to be given a choice in having video or physical consults. One health care participant opined that face-to-face consults are preferred by oncology patients for its “personal touch” (Staff 12, Nursing). It could be difficult for health care providers to build rapport through video consults (Table 3). A patient participant also expressed concerns of communication barriers during telehealth: “When you're using telehealth, is [there] time and the ability to ask … questions?” (Patient 10, age 46 years).

### Low uptake of online preanesthesia health assessment

Robust triaging of patients in the telehealth pathway is dependent on evaluation of the patient's health status. Some health care participants are concerned that low uptake of the online questionnaire at “about 25%” (Staff 4, PAS) could impact the quality of triage. Reasons proffered for the low uptake include obscure placement of the internet link within the text message sent to patients' mobile devices and technical issue affecting the flow of digital returns. To increase the uptake of health screening, health care participants suggested making both hard copies and the link to online questionnaires available to patients at the surgeons' clinics and training patient service associates to assist patients.

The reliability of patient responses to the health screening questionnaire was questioned (Table 3). In any case, a health care participant felt assured that patients would still be evaluated on the day of surgery and that serves as the safety net.

**d. Enablers of implementation**

Health care and patient participants made recommendations to facilitate the telehealth implementation plan, outlined in the following two subthemes.

### Skills and resources

Although the pandemic has accelerated the pace of digitalization, health care providers felt that having formal training in technological and communication skills for video consultation would be beneficial (Table 3).

### Receptiveness toward the new telehealth workflow

Health care and patient participants were, on the whole, open to explore a new telehealth workflow for preoperative evaluation. As long as adequate training is provided, health care participants at PAS had no objections conducting video consults (Table 3).

## Discussion

The study explores the perceptions and attitudes of key health care providers and elective surgery patients toward a telehealth model of care for preoperative evaluation at a tertiary women's hospital in Asia. Telehealth is convenient as it eliminates the need for travel, with accrued time- and cost-savings. Remote interactions also reduce the risks of COVID-19 transmission, while enabling care delivery during a pandemic. Our findings are in line with published studies that report high levels of satisfaction among patients and health care providers who participated in telehealth in the primary care and specialized settings.^25–29^

Both health care and patient participants in our study concur that telehealth may not cater to older patients and those who lack digital literacy. Research has shown that higher age, lower academic qualifications, and lower computer literacy can reduce a patient's willingness to adopt mobile health technologies,^30,31^ with younger and female patients emerging as early adopters of virtual consultations in one study.^31^ Given the demographic characteristics of patients at our institution (young and female), we are optimistic that a great proportion of our patients will be receptive to a new telehealth model of care. Physical consults could remain the primary mode of consultation for older patients and those who prefer a more personal touch.

Participants universally acknowledged the limitation of video consultations in providing for physical examination and opportunities in rapport building. Although this may be true to some extent, research also shows that communication skills for videoconferencing can be taught through a training program that is focused on communicating empathy and concern.^32,33^ The recent surge in telehealth initiatives has led to a call for training in telehealth competencies, focusing on digital communication and virtual interactions.^34,35^ This should be a priority of focus in our preimplementation work.

The perception that preoperative tests performed on the day of booking (>4 weeks in advance of surgery) may no longer be valid on day of surgery was a major barrier to telehealth workflow adoption among health care participants. It is, therefore, critical to address this in a pragmatic way. Fundamentally, there is value in performing early preoperative testing as it allows ample time for clinical abnormalities to be corrected. But what is a reasonable validity period for otherwise normal results? In a study of 970 patients, the probability of a change in previously normal preoperative tests was found to be only 1.7% (confidence interval [CI] 0.5–2.9) at 12 months in patients <50 years and 2.1% (CI 0.7–3.5) in those ≥50 years.^36^

Moreover, the likelihood for changes in test results was associated with increasing age (*p* = 0.009), higher surgical risk (*p* < 0.001), chemotherapy (*p* = 0.001), radiotherapy (*p* = 0.012), and comorbidities (*p* < 0.001).^36^ This suggests that the validity of normal preoperative tests could potentially be extended beyond 4 weeks without compromising patient safety. Accordingly, the institution's preoperative testing guidelines should be revised, with validity extended beyond 4 weeks.

The use of the NPT framework to underpin our qualitative analysis has surfaced key requisites for the new telehealth workflow and how health care providers and patients perceived the value of telehealth (coherence), identified the roles of health care providers and patients, including factors that inhibit (or enable) the embedding of preoperative telehealth consultations into routine practice (cognitive participation), and flagged the necessary collective actions needed to successfully operationalize telehealth in our setting. Specifically, the study has provided insights into the perceived impact of telehealth intervention on different groups of health care providers and the work they do.

It has also provided us with a measure of their level of cognitive participation that indicates whether health care providers could be engaged in collective action. Surgeons have expressed ambivalence in performing heart and lung examinations at the time of booking for surgery, citing concerns of missing a diagnosis and time pressures from running a busy clinic. The change in workflow for preoperative testing from scheduled appointments to unscheduled testing on day of booking also created tension among clinic managers and nurses who worried that existing capacities could be overwhelmed. The current level of coherence and cognitive participation among our health care participants suggests that greater buy-in and engagement is needed to gain their commitment for collective action.

Our findings suggest that an interprofessional working group should be formed to promote understanding and collaboration at different touch points to smoothen the workflow. The telehealth workflow will need to be redesigned, taking into consideration the barriers and enablers articulated. Data from this study will inform the planning of a pilot study to be conducted at one location to confirm feasibility before scaling to another location. The pilot would involve data collection to evaluate service fidelity at every touch point and quality of patient and health care provider experience.

There are limitations to this study. As it is conducted at a single institution with its female-only patient demographics, the findings cannot be generalized to other health care contexts. Findings of this qualitative study are influenced by the characteristics of the participants and their individual views. Characteristics of the research team members, including their identities, experience, disciplinary paradigms, and ability to think reflexively, could also affect the quality of analyses. Alternative perspectives were made possible by the diversity of our research team, which comprised senior doctors, a health services researcher and pharmacist, a senior health care research associate, and a medical student. The strength of this study lies in the use of an implementation science framework, the NPT, to guide the development and embedment of a new telehealth model of care. In doing so, we have identified real-world contextual barriers and will address the wide-ranging factors essential for successful implementation.

In conclusion, patients and health care providers acknowledge the benefits of and are receptive to having a telehealth model of care for preoperative evaluation. However, the proposed clinical workflows for preoperative testing and physical examination need to be redesigned, taking into consideration systems' constraints and capacities. Greater engagement is also needed to gain stakeholders' commitment for collective action.

## Supplementary Material

Supplemental data

Supplemental data

## Supplementary Material

Supplementary File SX

Supplementary File SY

## Abbreviations Used

KKH: KK Women's and Children's Hospital
NPT: Normalization Process Theory
OBGYN: Obstetric and Gynecological Service
PAS: Preadmission Service
RQ: research questions
SDA: same-day-admission
