# Supplementary material for: Perceptions and Attitudes of Patients and Health Care Stakeholders on Implementing a Telehealth Service for Preoperative Evaluation: A Qualitative Analysis
Source: Telemed Rep. 2023 Jun 26;4(1):156–65. doi: 10.1089/tmr.2023.0023 (PMC10523403; doi:10.1089/tmr.2023.0023)
Supplement: Supplemental data [file Suppl_DataSX.docx]

Supplementary File X: Interview guides for patients and healthcare providers
