# Supplementary material for: Perceptions and Attitudes of Patients and Health Care Stakeholders on Implementing a Telehealth Service for Preoperative Evaluation: A Qualitative Analysis
Source: Telemed Rep. 2023 Jun 26;4(1):156–65. doi: 10.1089/tmr.2023.0023 (PMC10523403; doi:10.1089/tmr.2023.0023)
Supplement: Supplemental data [file Suppl_DataSY.docx]

Supplementary File Y: Categories and Codes

| Category: **1 Advantages of telehealth workflow** | | |
| --- | --- | --- |
| Codes | Description | Source |
| *Convenient for patients* | Favoured by patients, due to the convenience, as they only have to come down if necessary e.g. saves a trip, saves time, no need to take leave from work | P,H |
|  | Pregnant women will benefit particularly from the convenience of teleconsultation. | H |
|  | Telehealth allows patients to continue with childcare responsibilities while receiving video consults. | P |
| *Convenient for care givers* | Accompanying care givers can participate virtually without commuting or taking leave of absence from work | H |
| *Saves costs for patients.* | Patients save cost due to current discount and transport cost. | H |
| *Reduces infectious risks in a pandemic* | Telehealth protects patients and staff from infectious risks during COVID-19. | H |
| *Eliminates physical waiting by patients and improves patient satisfaction* | Telehealth eliminate physical waiting at the hospital for patients and allow KK Hospital (KKH) to continue providing health services.  New workflow would greatly improve patient satisfaction scores and meet patients’ expectations.  Telehealth reduces the need for physical consult rooms which can be used for other purposes. | H  H  H |
| *Triage of patients in the telehealth workflow allows healthcare staff to focus on value-generating work* | The triaging system of the telehealth workflow allows doctors to focus on physical consults of high-risk patients, while patients with no or minor issues are assessed via video consults by nurses or junior doctors.  With more patients adopting telehealth, staff can spend more time on value-generating work as they do not need to manage patients physically. | H  H |
| *Telehealth may allow consults to be conducted more punctually* | Patients are optimistic that their video consults can commence punctually. Consults can also be recorded for playback.  Telehealth allows the bypassing of physical barriers, allowing patients to be more punctual, improving the flow of the schedule. | P  P |
| *Improves time management and efficiency of clinic* | Telehealth may allow more patients to be processed more efficiently, due to better time management. | P, H |
| *Early pre-op investigations in the proposed new workflow may detect abnormalities earlier* | New workflow could be advantageous by encouraging earlier investigation and allowing the subsequent consult to be more informed. | H |
|  | Early detection of abnormalities through early pre-operative investigations can allow early treatment, and repeat tests may not be needed before surgery. | H |
| *Advantages of video consults over phone calls* | Video consults allow ‘face-to-face’ communication, improving patients’ experience and allowing anaesthetists to get a sensing of the patient’s emotions and response. | H |

Legend - H: healthcare participant P: patient participant

| Category: **2 Physical examination must be performed by surgeon on day of listing for surgery** | | |
| --- | --- | --- |
| Codes | Description | Source |
| *Physical examination must be conducted by a doctor before day of surgery* | A thorough preoperative physical examination must be done by a doctor, whether at the surgeon’s clinic or Preadmission Service (PAS). | H |
|  | Surgeons can be entrusted to perform physical examination and detect obvious abnormalities which are clinically important. Scans mandated for vulnerable patients prevent reliance on physical examination | H |
| *Requirement of physical examination should be clearly stated* | Physical examination during preoperative assessments should include heart and lung auscultation, preliminary examination of dental hygiene and an assessment of obesity. | H |
| *Under the new workflow, there is a need to take vital signs once patients are listed for surgery* | Currently, vital signs are taken only at the first medical visit; not taken for follow-up visits. | H |

Legend - H: healthcare participant P: patient participant

| Category: **3 General requisites of the new telehealth workflow** | | |
| --- | --- | --- |
| Codes | Description | Source |
| *Punctuality of patients affects efficiency of virtual consults* | Punctuality of patients may affect efficiency of telehealth.  Appointment timings must be strictly adhered to in new workflow, giving impetus to follow schedule and not delay subsequent consults. | H  H |
| *There is a need to create a planned admission before preoperative testing.* | A planned admission must be created before preoperative testing at the surgeon’s clinic so that investigation costs can be included in the surgical bill. | H |
| *Patients should be given the option for video or physical consults* | Patients should be given the option for video or physical consults | P |
| *Telehealth should be conducted via secure network* | Telehealth should be conducted via secure hospital and home networks | P |
| *Telehealth should be conducted in a private setting* | As confidential or sensitive issues may be discussed, telehealth should be conducted in a private environment for both healthcare staff and patients. | H |

Legend - H: healthcare participant P: patient participant

| Category: **4 Preop investigations to be performed on day of listing for surgery** | | |
| --- | --- | --- |
| Codes | Description | Source |
| *Preoperative investigations have limited validity* | Preoperative investigations are valid for 30 days. If surgery is postponed for more than 30 days, patients have to repeat their blood tests.  Patients may be unwilling to incur additional cost of repeat blood tests. | H  H |
|  | Preoperative investigations done 6 to 8 weeks prior is not a limitation as they can be accepted in *well or stable patients.*  If necessary, blood tests can be repeated on the day of surgery e.g. in dialysis patients. | H |
|  | Patients who present with low haemoglobin on surgery day may not necessarily have their surgery cancelled but would require a valid cross match and repeat tests on day of surgery. | H |
|  | When patients are undecided about the date of surgery, the validity of the preoperative investigations can be an issue. | H |
|  | Validity of preoperative investigations may be affected by waiting time for surgery which is of 1 to 3 months’ duration for subsidized patients with benign conditions. | H |
| *Surgeons should not book surgery dates for patients who are undecided.* | Patients who are undecided about surgery should be given time for consideration before committing to a date to reduce wastage of slot. | H |
| *Preop investigations may become invalid in patients with longer wait times to surgery* | Preop blood tests could remain valid till date of surgery for private patients, but less likely for subsidized patients due to longer waiting time. | H |
| *The validity period of investigations could vary according to medical risk of patients (which is aligned to triaging criteria)* | Patients triaged to Telehealth are of no or low risk and hence, a longer validity period may be acceptable for their preoperative investigations. | H |
| *A major contributory factor to long waiting time for surgery is the lack of OT slots during OT renovation* | The lack of operating room slots, and not patient preference, is the primary reason for long waiting times to surgery. | H |
| *Some patients may cancel or change the date of the surgery* | A small percentage of patients are uncertain about their surgery and may cancel or postpone the surgery. | H |
| *Doctors’ orders of preoperative investigations* | Phlebotomists depend on clarity of doctors’ orders, currently vetted by PAS nurses.  Doctor’s orders on electronic health records need to be verified by PAS nurses as they may be unclear, missing or duplicated. | H |

Legend - H: healthcare participant P: patient participant

| Category: **5** **Robust triaging criteria is necessary** | | |
| --- | --- | --- |
| Codes | Description | Source |
| *Importance of having robust criteria in triaging patients to telehealth* | Acknowledges the importance of having robust criteria so that patients with major medical issues can be reviewed in-person. | P,H |
|  | Clear and robust triaging guidelines allows majority of patients’ needs to be catered for. | H |
| *Triaging criteria for physical consults* | American Society of Anesthesiologists’ class 3 patients with propensity to develop complications and those with acute cardiovascular conditions should be reviewed in person. | H |
|  | Patients with abnormal investigation results should have physical consults. | H |
|  | Patients with abnormal investigations should be recalled for intervention before surgery. | H |
|  | Patients with more significant medical issues should be assessed in person. | P |
| *Video consults or phone consults are feasible for well patients.* | Video consults and phone consults are feasible for well patients.  Patients benefit extensively as long as triaged appropriately for the telehealth and are able to show evidence of home monitoring. | H  H |
| *Having patients screened at PAS in the current workflow assures surgeons that cases will not be cancelled on surgery day.* | Having their patients screened and optimised in-person at PAS gives surgeons assurance that patients will not be cancelled on surgery day - which is their primary concern.  Abnormal results could be known at the time of PAS visit, facilitating appropriate optimization and reducing the risk of case cancellation. | H  H |
|  | Rarely, patients who are screened as normal at PAS can still be postponed if an abnormality is detected on surgery day. | H |
| *The new workflow is safe and robust for preanaesthesia evaluation* | As there is still one crucial final evaluation of the patient on the day of surgery, regardless of the type of platform of the patient’s previous consult, the new workflow is safe and robust. | H |
| *Disagreement on the patient’s level of triage could occur between preoperative and intraoperative anaesthesia providers* | Telehealth triage criteria must be discussed and broadly accepted by all stakeholders, with staff being trained to implement it, to prevent disagreement in management | H |
| *Reliability of patient self-administered health assessment* | There is concern that patient responses to online health assessment do not corroborate with doctor’s medical history-taking and this could affect quality of care. | H |
|  | There is concern that patients do not declare their medical conditions accurately. | P |

| Category: **6 Barriers and challenges in performing physical examination** | | |
| --- | --- | --- |
| Codes | Description | Source |
| *Surgeons may be reluctant to perform physical examination for patients on day of listing* | Requirement for physical examination will need buy-in from surgeons who may be reluctant to perform heart and lungs examination. | H |
|  | Surgeons may be reluctant to perform certain aspects of examination. | H |
|  | Surgeons can be convinced to perform physical examination and preoperative testing if they recognize the convenience it brings to their patients. | H |
|  | Surgeons may be concerned that they have lost touch in performing physical examination of heart and lungs | H |
|  | Junior doctors e.g. residents, may be more familiar and thus, more confident in conducting physical examination, if a stethoscope is available | H |
|  | More senior doctors, such as consultants and senior consultants, may be more reluctant to perform physical examinations | H |
|  | Doctors may not have the time to conduct physical examination due to high patient load and longer individual consultation time, especially in busy clinics. | H |
|  | Heart and lung examinations are not consistently performed by most surgeons. | H |

Legend - H: healthcare participant P: patient participant

| Category: ***7* Barriers and challenges in using teleconsultation (for patients and staff)** | | |
| --- | --- | --- |
| Codes | Description | Source |
| *Not tech savvy* | Not competent with technology or have limited access to internet  e.g. not familiar with smart phones, computer and Wi-Fi setup | P, H |
|  | Patients who are not competent with technology  may not know how to respond to online health screening questionnaire. | H |
| *Language barrier* | Those who are not tech savvy could be illiterate as well, thus having a poster to guide them would not suffice. They would require someone to help them. | P |
|  | Education materials sent to patients are in English only. Multi-lingual instructions are necessary. | H |
| *Challenges for Older Adults* | Older patients may need help from family members with telehealth as they are not competent with technology. | H |
|  | Older patients may not even have mobile devices. | P |
|  | Instructions on telehealth are only in English and some older adults may not be able to read English. | H |
|  | Older patients may be resistant as they are more assured by physical consults. | H |
|  | Patient Service Associates working in Preadmission Service are of older age and may require more technological training. | H |
|  | Older patients who lack technological skills should go through the traditional workflow and be reviewed in person instead. | P |
| *Caregiver support of older patients* | Unaccompanied older patients may fail to retain verbal instructions or understand written instructions in English.  Some older patients may not want to bother their children with the interpretation of written instructions. | H  H |
| *Telehealth does not benefit healthcare staff directly who also have to learn new technological skills* | Telehealth confers no time savings for healthcare staff who also need to learn the technological set-up for telehealth.  Staff do not benefit - it is merely a change in the model of care. | H |
| *Patients may be concerned that virtual consultation may provide less thorough care* | Patients may be resistant to telehealth as they are concerned that their care may be less thorough. | H |
| *Surgeons’ and patients’ cost concerns regarding virtual consultation* | Surgeons may be concerned of the cost of telehealth, and patients may feel that the telehealth consultation fees should be lower than that of in-person consultation. | H |
|  | Patients are not likely to be bothered about the cost of telehealth for preoperative care as it is incorporated into the inpatient surgery fees. | H |
|  | Patients may refuse to pay for their telehealth consults if they perceive that the consult does not add value to their care. | H |
| *Some doctors may require support to perform virtual consults effectively* | Some doctors may not be fully competent using Zoom and need training. | H |
| *Phone calls to patients may be regarded as scam calls* | Patients may mistake hospital phone calls as scam calls and refuse to answer. It is necessary to pre-empt via them via SMS texts or email. | H |
| *Patients’ expectation to have medical certificates (MC) for video consults* | Patients undergoing telehealth may expect to have a medical certificate (MC) issued. | H |
| *Patients’ preference for the type of consultation may depend on whether they can get MC* | Patients may prefer in-person consultation, if medical certificate (MC) is not issued for telehealth consults. | H |
| *Technical issues for telehealth consultation* | Wi-Fi connectivity of the location could be an issue. | H |
|  | Ease of patient logging in for telehealth could be an issue. | H |
| Category: **8 Low uptake of online preanaesthesia health assessment** | | |
| Codes | Description | Source |
| *Low uptake of current online health assessment questionnaire* | Current uptake of online questionnaires is not high.  Only 25% of patients complete the online health assessment questionnaire. | H |
|  | Current uptake of online questionnaires is 40-50%. | H |
| *Obscure link for health assessment questionnaire on SMS not noticeable by busy patients* | Placement of web link for health assessment questionnaire at the end of the SMS message may be the reason why the link is not noticed by patients or that patients may fail to read the entire SMS and therefore, do not click on the link. | H |
| *No reminders sent* | No reminders are sent to patients to complete the online health assessment questionnaire. | H |
| *Patients do not respond despite reminders sent* | Patients do not usually respond to reminders sent. | H |
| *Patient responses may fail to flow into the system* | There could be failure of online patient responses to flow into the electronic health records. | P |

Legend - H: healthcare participant P: patient participant

| Category: **9** **Enablers of Telehealth Workflow** | | |
| --- | --- | --- |
| Codes | Description | Source |
| *Technological savviness* | Telehealth adoption will be higher among younger and more technologically savvy patients. | H |
|  | A survey at Clinic C shows that 100% of the patients have a smart phone, hence the adoption rate for telehealth is likely to be high. | H |
| *The acceleration of digitization during the pandemic has improved people’s technological competence and increased adoption of telemedicine* | The pandemic has increased the rate of adoption of telemedicine, especially among the technologically-savvy. | H |
|  | Patients prefer to have virtual consults in this COVID situation, especially well patients. | H |
|  | The pandemic has shown that people embrace technology in monetary transactions and other businesses *(e.g. TraceTogether, Singpass*), thus they will likely embrace the use of technology in healthcare as they *have become more familiar with technology too.* | H |
| *Nurses can be trained to perform video consultation* | A standard procedure must be undertaken to train nurses to conduct airway examinations on telehealth platforms, and document their certification. | H |
|  | Nurses should undergo technological training to familiarise with telehealth platform. | H |
|  | Nurses also may need to be trained to be better at understanding and sensing patients’ needs on video consults. | H |
| *A telehealth workflow is already implemented for follow-up patients at PAS with positive staff and patient experience* | A telehealth workflow has been implemented for follow-up patients at PAS and patients’ questions were easily addressed. | H |
| *No technical issues for telehealth consultation* | No technical issues are reported with early telehealth patients for PAS follow-up | H |
|  | Assistance was provided by departments who are more experienced in conducting video consults. | H |
| *To increase uptake of online health assessment questionnaire by providing clear instructions and getting Patient Service Associates to administer it at SOC.* | Uptake of online questionnaire can be increased by providing clear instructions for patients to embark at their homes.  Hard copy and online health assessment questionnaires can be administered to patients at surgical clinics by trained Patient Service Associates, while patients are waiting to be processed. | P |

Legend - H: healthcare participant P: patient participant

| Category: **10** **Factors that will increase adoption of virtual consults by patients** | | |
| --- | --- | --- |
| Codes | Description | Source |
| *Clear explanation given to patients on how to connect* | Patients can be convinced to use telehealth if clear explanation is given. | H |
|  | Patients should be sent a link and a step-by-step guide for ease of connection to telehealth. | P |
| *Patients are assured that their care is not compromised by the telehealth workflow.* | If patients are reassured of a physical consult when abnormal findings are detected, they will be more receptive towards the new telehealth workflow. | H |
| *Patients assessed by own doctor* | Patients are amenable to video consults as long as they are conducted by their own doctor | H |

Legend - H: healthcare participant P: patient participant

| Category: **11** **Attitude of healthcare staff towards new Telehealth Workflow** | | |
| --- | --- | --- |
| Codes | Description | Source |
| *Ambivalence of surgeon’s clinic staff towards new workflow* | Some surgeon’s clinic staff may not be receptive to the new workflow | H |
|  | New workflow of physical examination and investigation at surgery clinics is deemed to be manageable by surgeons. | H |
|  | Nurses are willing to carry out additional blood taking at surgeon’s clinics as per new workflow. | H |
| *No objections to conducting teleconsultation in PAS* | As long as staff are trained, nurses at PAS have no objections conducting telehealth at PAS which is already implemented for post-referral anaesthesia consults. | H |
| *Willingness of healthcare staff to learn technological skills for telehealth* | Healthcare staff feel that technological skills of telehealth can be learned easily.  Acknowledges the importance of learning technology, given its prevalence today | H |

Legend - H: healthcare participant P: patient participant

| Category: **12 Perceived impact of new Telehealth Workflow on SOC and PAS** | | |
| --- | --- | --- |
| Codes | Description | Source |
| *New workflow will increase processing time at surgeon’s clinic* | New workflow may increase processing time for nurses at surgeon’s clinic due to unpredictability of workload for preoperative testing. | H |
|  | Waiting time for subsidized patients at the surgeon’s clinic is already long currently.  New workflow may increase processing time for doctors at the surgeon’s clinics. | H |
|  | If investigations can be done only after listing of surgery is confirmed, patient service time at the surgeon’s clinics may be prolonged. | H |
| *Private patients attending the surgeon’s clinics are able to participate in the new workflow* | Surgeon’s clinics attending to private patients may be able to cope with the new workflow if workload for preoperative testing is predictable. | H |
| *Insufficient infrastructure e.g. space, at surgeon’s clinics* | Nurses can upskilled to perform physical examinations, but treatment rooms at Surgeon’s clinics have insufficient space. | H |
|  | Infrastructure of surgeon’s clinics is space-limited and it may be congested for patients to undergo investigations there. | H |
|  | Some surgeon’s clinics have only one phlebotomy station and may not be able to cope with the additional workload. | H |
| *Workload for taking blood at surgeon’s clinics would likely remain unchanged, even with implementation of the new workflow* | Although surgeon’s clinics are currently performing phlebotomy for Ambulatory Surgery patients, taking blood for Same-Day-Admission surgery patients will probably not increase the work load very significantly. | H |

Legend - H: healthcare participant P: patient participant

| Category: **7** **Current constraints at SOC and PAS** | | |
| --- | --- | --- |
| Codes | Description | Source |
| *Current workload for surgeon’s clinic staff is heavy* | Current workload at surgeon’s clinic is heavy due to multi-tasking e.g. blood taking, giving medications and doing other investigations | H |
|  | Clinic “C” can list 100-120 patients for elective surgery every month. | H |
| *There is limited staffing at certain surgeon’s clinics* | Staffing at surgeon’s clinics is limited to 3-4 nurses and 1 phlebotomist. | H |
| *Surgeon’s clinics receiving private patients have high workload* | Surgeon’s clinics receiving private patients have high workload due to subspecialty patient needs. | H |
|  | Surgeon’s clinics receiving private patients are now serving subsidized patients too and hence, have an increased workload. | H |
| *Inappropriate use of PAS services for ambulatory surgery cases* | There is inappropriate use of PAS services due to ward staff being unsure about current workflow. For example, patients listed for ambulatory surgery are asked to come to PAS for blood test, when they should actually diverted to the surgeon’s clinic based on current workflow. | H |

Legend - H: healthcare participant P: patient participant

| Category: **8 Brainstorming of alternative workflow** | | |
| --- | --- | --- |
| Codes | Description | Source |
| *Brainstorming of alternative workflow to send walk-in patients to PAS for blood tests* | There are objections to receiving patients as unscheduled walk-ins from surgeon’s clinics to PAS due to limited manpower and unpredictability of workload at PAS.  There is time constraint in processing a patient at PAS, including time spent retrieving “lost” Scanned Medical Record (SMR). | H |
| *Having mobile phlebotomists who move between PAS and surgeon’s clinics* | Concept of having mobile phlebotomists who move between PAS and surgeon’s clinics but this workflow may face physical constraint at the surgeon’s clinics. | H |
|  | Concept of having a phlebotomist at every floor level to service the surgeon’s clinics on that level. | H |
| *Upskill surgeon’s clinic nurses to perform physical examination at the surgeon’s clinics* | Nurses could be upskilled to help with conducting physical examinations if doctors do not have enough time to attend to all. | H |
|  | While nurses may be upskilled to help conduct physical examination, they have multiple responsibilities and may not have enough time either, if it takes more than 15 minutes per patient. | H |
| *Incorporation of workflow for the advance practice nurse-led clinic into the new telehealth workflow* | The advance practice nurse-led clinic workflow should be incorporated into the new telehealth pathway to save patients from another trip. | H |
|  | Patients who are identified to require consultation at advance practice nurse-led clinics could be directed there on the same day as attendance at surgeon’s clinic to save them another trip. However, that will require boosting of staffing and infrastructure to cope with walk-in demands. | H |
| *Physical examination performed at primary care settings* | Getting patients examined at primary care settings is an alternative but it involves an additional visit. | H |
| *Setting up a common station for physical examination and phlebotomy at surgeon’s clinics* | A station may be set up at the surgeon’s clinics, dedicated to physical examination and phlebotomy of patients who are booked for surgery. | H |
| *PAS may be able to take on the workload of physical examination* | The task of performing physical examination could be directed at the PAS, but some changes are necessary to ease this workflow e.g. data analytics to assess daily workload. | H |
| *Accessibility and directions to PAS* | There is concern that patients may not be able to find their way to the PAS from surgeon’s clinics. | H |
| *Physical examination can be performed by trained advanced practice nurses (APNs).* | Physical examinations can be performed by trained APNs. | H |

Legend - H: healthcare participant P: patient participant

| Category: **9 Limitations of virtual consultation** | | |
| --- | --- | --- |
| Codes | Description | Source |
| *Telehealth may miss the detection of medical conditions, unlike in-person.* | Telehealth has its limitations in that some details, including body language of patients, may be lost, affecting ability to assess patients holistically. Less effective than in real life. | H |
|  | Telehealth may miss the detection of conditions if physical examination or physical contact is warranted. | H |
| *Video consultations lack personal touch.* | Oncology patients may still prefer physical consultations as they need emotional support and personal touch which is lacking in video consults. | H |
| *Telehealth consultation is more ideal for consultation and counselling* | Telehealth is more ideal and acceptable for consults of a consultative and counselling nature. | H |

Legend - H: healthcare participant P: patient participant

| Category: **10** **Potential barriers in Telehealth Workflow that can be circumvented** | | |
| --- | --- | --- |
| Codes | Description | Source |
| *Referrals can be made via telehealth* | E-referrals can be made via telehealth for patients who require referral to medical subspecialities. | H |
| *Airway examination can be performed virtually* | With good lighting and adequate image resolution on video consultation platforms, it is feasible to perform airway examination via telehealth. | H |
| *Hence, telehealth is acceptable for patients with anticipated or known difficult airway* | Telehealth is acceptable for patients with difficult airway as virtual airway assessment can be done and plan of management discussed through telehealth.  Airway examination is also repeated on the day of surgery. | H |
| *E-financial counselling could be incorporated into telehealth* | Many patients do not complete financial counselling before op day, thus incorporating it into the telehealth workflow as a package could be adopted. | H |
| *Expiry of surgical consent validity* | Surgical consent is valid for 3 months and can be re-validated on day of surgery. | H |
| *Establishment of rapport and engagement with patients* | Video consultation allows eye contact for establishment of engagement and rapport with patients. | H |
| *Preoperative instructions sent my mail may not be received or opened.*  *Preoperative education materials can be shared prior to and during video consults.* | Instructions sent by mail may not be received/opened by patients.  Preoperative education materials can be shared with patients in advance, with clarification during video consults. | H  H |
| *The new telehealth workflow should make provisions for bowel preparation solutions and perioperative chlorhexidine wash to be dispensed on the day of listing for surgery.* | Currently, patients receive chlorhexidine wash from PAS and bowel preparation solutions from the hospital pharmacy. In the new workflow, these may have to be dispensed on the day of booking for surgery. | H |

Legend - H: healthcare participant P: patient participant

| Category: **11 Telehealth implementation plan** | | |
| --- | --- | --- |
| Codes | Description | Source |
| *Buy-in from stakeholders can be obtained through data collection and sharing of good outcomes* | Data can be shared to demonstrate improved efficiency of new workflow and improved patient and healthcare provider satisfaction scores. | H |
| *Operational details of implementation* | There is suggestion to conduct a pilot in the one or two surgeon’s clinics, get buy-in from healthcare providers and fine tune the new workflow before scaling to other clinics. | P |
| *Create patient awareness of the new model of care to increase adoption* | Patients’ awareness and uptake of the telehealth model of care can be increased by publicity measures e.g. brochures and recommendation from their surgeons. | P |
| *Create awareness of telehealth workflow among junior rotating doctors* | Junior rotating doctors lack awareness of the current telehealth workflow for follow-up patients at PAS.  Unlike advanced practice nurses, rotating doctors currently lack awareness of the availability of telehealth for follow-up patients at PAS. | H |
| *Feasibility of new workflow depends on collaboration between stakeholders* | New telehealth workflow would be feasible if there is effective collaboration between different healthcare providers servicing the different patient touch points. | H |

Legend - H: healthcare participant P: patient participant
